# Cryo-EM structure of activated bile acids receptor TGR5 in complex with stimulatory G protein

**DOI:** 10.1038/s41392-020-00262-z

**Published:** 2020-08-03

**Authors:** Geng Chen, Xiankun Wang, Yunjun Ge, Ling Ma, Qiang Chen, Huihui Liu, Yang Du, Richard D. Ye, Hongli Hu, Ruobing Ren

**Affiliations:** 1 Kobilka Institute of Innovative Drug Discovery, School of Life and Health Sciences, The Chinese University of Hong Kong, 518172 Shenzhen, Guangdong P.R. China; 2 School of Life Sciences, University of Science and Technology of China, 230026 Anhui, P.R. China; 3 Warshel Institute for Computational Biology, The Chinese University of Hong Kong, 518172 Shenzhen, Guangdong P.R. China

**Keywords:** Structural biology, Biochemistry

**Dear Editor,**

Takeda G protein-coupled receptor 5 (TGR5), also known as G protein-coupled bile acids (BAs) receptor 1 (GPBAR1),^1^ belongs to the class A GPCR subfamily. The major TGR5-dependent actions of BAs include maintaining energy homeostasis, regulating glucose/lipids metabolism, as well as immunosuppressive properties.^2^ TGR5 is identified as a potential therapeutic target for protecting hepatocytes from bile acid overload, preventing atherosclerosis, and inhibiting macrophage inflammation due to its critical role in bile acid sensitization. Thus, elucidation of structural characteristics of TGR5 and its activation mechanism would benefit the discovery of therapeutic drugs for these metabolic disorders.

TGR5 activity is governed by endogenous unconjugated or glycine-/taurine-conjugated primary and secondary BAs, semisynthetic derivatives, and some synthetic nonsteroid molecules (Fig. 1a, left panel). Here we report the near-atomic resolution cryo-EM structure of activated TGR5 in complex with the synthetic nonsteroid agonist 23H^3^ and G_s_ protein (Fig. 1b, Supplementary Fig. 1a). For cryo-EM structure determination, we engineered human TGR5 protein (Supplementary Fig. 1b, c). The modified TGR5 retains comparable nanomolar efficacy to several agonists as the wild-type receptor (Fig. 1a, right panel). Vitrified complexes were imaged and processed to yield the map of TGR5-G_s_ complex at an overall resolution of 3.9 Å (Fig. 1b, Supplementary Figs. 2–3, and Table 1). Backbones of transmembrane helices (TMs) are resolved as well as residues with bulky side-chains. The TGR5 interfaces with G_αs_, including α5-helix of G_αs_, were also well defined (Supplementary Fig. 4).

**Fig. 1:**
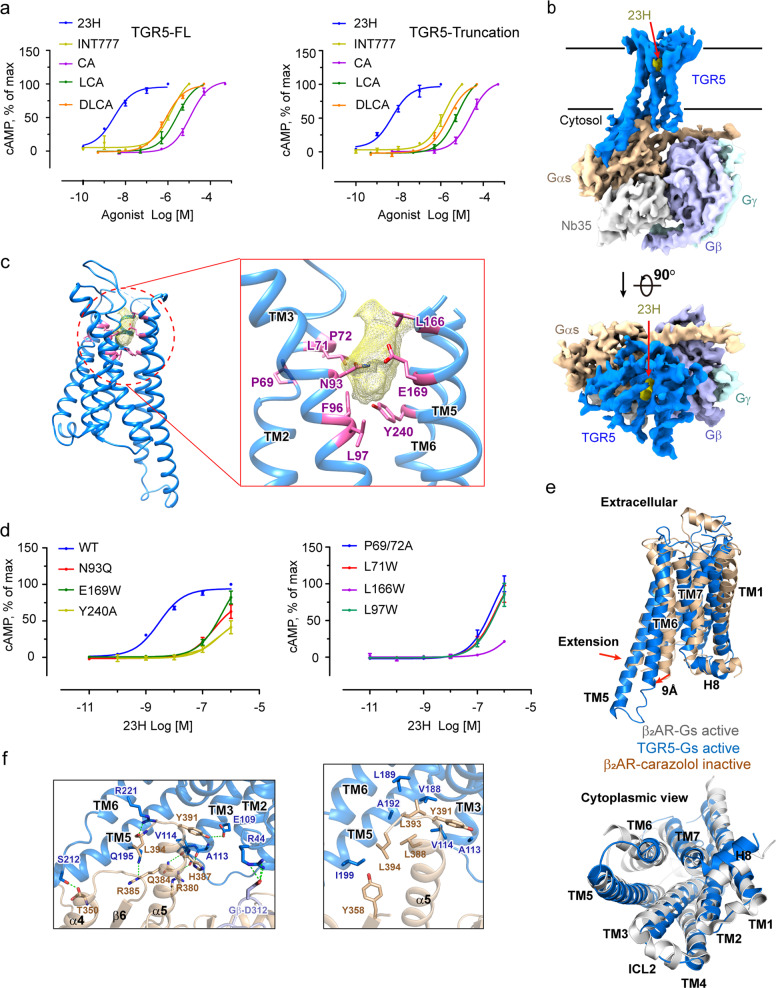
Structural and biochemical studies of TGR5-Gs complex. **a** cAMP response of full-length and truncated TGR5 with compounds 23H, INT77, CA, LCA, and DLCA. cAMP responses are shown as percentages of the maximum response of each ligand. The data represent means ± S.E.M. (*n* = 3–5) and most error bars are within the dimensions of the data points. **b** Cryo-EM structure of TGR5-Gs complex. TGR5, G_αs_, G_β_, G_γ_, Nb35, and 23H are shown in blue, wheat, light blue, light green, grey, and yellow, respectively. **c** Residues in TGR5 that involve in 23H binding. Density of 23H is shown in yellow. Residues that might involve in 23H binding are shown in pink. **d** cAMP responses of mutant TGR5. These mutational TGR5 reduced agonist potency by two order compared with wild-type. The corresponding pEC50 is shown in supplementary Table 2. cAMP responses are shown as percentages of the maximum response of the WT. The data represent means ± S.E.M. (*n* = 3–5). WT data were not shown on panel **b** (right panel) because all the mutations were tested at the same time. **e** Comparisons of active TGR5 (blue) with active (grey) and inactive (wheat) β_2_AR. **f** Interface of TGR5 with Gs protein. Residues in TGR5 are shown in blue and residues in Gαs are shown in wheat. D312 in Gβ is shown in light blue

The density representing 23H was observed adjacent to the extracellular base of TM3, TM5, and TM6 (Fig. 1b and Supplementary Fig. 5a). Due to the limited quality of density map, 23H cannot be precisely modeled in the structure. A sketchy docking was applied to confirm that, the omitted density in the putative TGR5 orthosteric site can accommodate the entire 23H (Supplementary Fig. 5b). By structural analysis combining with intracellular cAMP measurement studies, we extensively screened and identified clusters of residues in the orthosteric site that are critical for 23H induced TGR5 activation (Fig. 1c, d, Supplementary Fig. 5, and Table 2). Within the orthosteric site, TGR5 established interactions with 23H through residues on TM2, TM3, TM5, and TM6. L71W^2.60^ decreased the potency of 23H by two orders of magnitude, indicating the possible stereo clash between the bulky side-chain and 23H. P69^2.58^/72A^2.61^ double mutation also reduced the potency of 23H by two orders of magnitude, suggesting that this unique PXXP kink located on the cytosolic half of TM2 may stabilize 23H bound conformation of the orthosteric site. N93Q^3.33^ decreased the potency of 23H by two orders of magnitude, indicating possible hydrogen bond formation between N93^3.33^ and 23H. F96A^3.36^ caused reduced agonist potency with 23H by two orders of magnitude, which might be partly contributed by reducing the hydrophobic interaction with 23H. Bulky side-chain residues substitution of L97^3.37^ to Trp and Phe reduced agonist potency by two orders and one order of magnitude, respectively, raising the possibility that bulky side-chains may have the stereo clash with 23H indicative of hydrophobic interaction with 23H. L166W^5.40^ and E169W^5.43^ caused reduced cAMP response, indicating that bulky side-chains may clash with 23H. Y240^6.51^ to Ala but not Phe reduced agonist potency by two orders of magnitude, indicating hydrophobic interaction between Y240^6.51^ and 23H. Other residues, which reduced the potency of 23H by one order of magnitude, are described in Supplementary Text.

23H has divergent chemical structure comparing to bile acids yet initiate convergent G_s_ coupling and signal transduction through TGR5. To unveil the molecular mechanism of convergence, we examined the potency of agonist LCA to TGR5 mutants in cAMP assays (Supplementary Fig. 7, and Table 2). Consistently, L71W^2.60^, L74W^2.63^, L166W^5.40^, E169W^5.43^, and Y240A^6.51^ compromised the potency of LCA. Y89A^3.29^, which have little effect on the potency of 23H, also decrease the potency of LCA by one order of magnitude. W75^2.64^, as a “lid”, made the orthosteric binding site occluded. However, W75A^2.64^ did not affect potencies of 23H and LCA. Notably, F96A^3.36^ compromised the potency of 23H but not of LCA. These data suggested that 23H and LCA to a great extent shared the same binding site but had slight differences in recognition details.

TGR5 possesses the same fold of class A GPCRs. Since TGR5 and β_2_AR share an overall 22% sequence identity (Supplementary Fig. 8), structural alignments of active TGR5 with that of inactive (PDB code: 2RH1) and active (PDB code: 3SN6) β_2_AR^4^ were performed, respectively (Fig. 1e and Supplementary Fig. 9). In the superposition of active TGR5 and inactive β_2_AR, the overall r.m.s.d is 2.9 Å over 145 residues majorly located on the TM region. The N-terminus of TM6 in TGR5 swing outward about 9 Å (the distance between C_α_ of residue K267 in TGR5 and the corresponding residue R216 in β_2_AR), resulting in the elevation of intracellular terminal of TM6 for G_αs_Ras interaction. Two helical turns extension of TM5 helix, which contributed to the interaction between TGR5 and G_αs_Ras, was observed (Fig. 1e, upper panel). These structural features are coincident with previous studies in β_2_AR activation. Viewing towards the membrane plane from the intracellular side, the TMs at cytoplasmic half of activated TGR5 and β_2_AR assume similar topology (Fig. 1e, lower panel). Thus, both TGR5 and β_2_AR form a similar cavity recognizing the C-terminal of the α5-helix of G_αs_Ras domain.

The structural superposition of TGR5-G_s_ with β_2_AR-G_s_ reveals that the G protein adopts almost identical conformation (Supplementary Fig. 10). The main differences of G_αs_ between the two complexes are located at β2, β6, α4, and N-terminal of α5 in G_αs_Ras. The main differences of G_βγ_ are located at some β sheets in G_β_. The total buried interface of the TGR5-G_α_Ras, which is mediated by extensive hydrogen bonds and hydrophobic interactions, is about 841 Å^2^. This interface is majorly composed by TM3/5/6, ICL1/3 of the TGR5, and α4/5 helices, β6 strand of G_α_Ras domain. Most of the residues involved in TGR5 interaction are in the carboxyl-terminus of α5-helix of G_α_Ras, such as Q384, H387, Y391, L393, and F394. It is consistent with the observation in β_2_AR-Gs interaction (Fig. 1f), suggesting the conserved G_s_ binding and activation mechanism.

Sequence analysis revealed that several TGR5 residues involved in the interaction were identical to that in β_2_AR, including E109^3.49^ (the most highly conserved amino acids E/DRY, which are located at the cytoplasmic ends of TM3), A113^3.53^, V114^3.54^, V188^5.62^, A192^5.66^, and Q195^5.69^ (Fig. 1f and Supplementary Fig. 7). It is worth mentioning that D312 in G_β_ forms hydrogen bonds with R44^ICL1^ of TGR5 (Fig. 1f), which was coincident with G_s_-coupled peptide activated class B GLP-1 receptor^5^ but not in β_2_AR. This suggested that other than stabilizing the N-terminal α helix of G_αs_, G_β_ might also involve in receptor binding. Besides, Nb35 binds to the interface between G_β_ and G_αs_Ras to stabilize the complex for structure determination (Fig. 1a).

In summary, our studies on TGR5-G_s_ complex structure and mutagenesis analysis revealed the agonist binding mode of TGR5 indicating the convergent activation mechanism, in which the orthosteric binding site could recognize distinct ligands and accommodate the receptor activation. The slight differences in detailed recognition of 23H and LCA will also shed light on the development of therapeutics with improved efficacy and specificity. We firmly believed that TGR5 is a proper prototype on the mechanistic understanding of other GPCRs sensing steroids.

## Supplementary information

Supplementary Information

Supplementary Figure 1

Supplementary Figure 2

Supplementary Figure 3

Supplementary Figure 4

Supplementary Figure 5

Supplementary Figure 6

Supplementary Figure 7

Supplementary Figure 8

Supplementary Figure 9

Supplementary Figure 10

Supplementary Figure 11

## Data Availability

All relevant data are available from the authors and/or included in the manuscript. Atomic coordinates and EM density maps of the human TGR5 have been deposited in the Protein Data Bank (PDB code: 7BW0) and the Electron Microscopy Data Bank (EMDB code: EMD-30221), respectively.
