# Supplementary material for: Cryo-EM structure of activated bile acids receptor TGR5 in complex with stimulatory G protein
Source: Signal Transduct Target Ther. 2020 Aug 3;5:142. doi: 10.1038/s41392-020-00262-z (PMC7400742; doi:10.1038/s41392-020-00262-z)
Supplement: Supplementary file 1 — Supplementary Information [file 41392_2020_262_MOESM1_ESM.docx]

**Supplementary information for**

**Cryo-EM structure of activated bile acids receptor TGR5 in complex with stimulatory G protein**

Geng Chen^1,2*^, Xiankun Wang ^1,2*^, Yunjun Ge^1*^, Ling Ma^1,2*^, Qiang Chen ^1,2^, Huihui Liu^2,3^, Yang Du^1^, Richard D. Ye^1^, Hongli Hu^1#^, Ruobing Ren^1#^

^1^ Kobilka Institute of Innovative Drug Discovery, School of Life and Health Sciences, the Chinese University of Hong Kong, Shenzhen, Guangdong 518172, P.R. China

^2^ School of Life Sciences, University of Science and Technology of China, Anhui 230026, P.R. China

^3^ Warshel Institute for Computational Biology, the Chinese University of Hong Kong, Shenzhen, Guangdong 518172, P.R. China

* These authors contribute equally

# To whom correspondence should be addressed. Email: [honglihu@cuhk.edu.cn](mailto:honglihu@cuhk.edu.cn) and [renruobing@cuhk.edu.cn](mailto:renruobing@cuhk.edu.cn)

Ruobing Ren

School of Life and Health Sciences, the Chinese University of Hong Kong, Shenzhen, Tu H.L. building, 7^th^ Floor, Longxiang Road, Shenzhen, Guangdong 518172, China.

Email: renruobing@cuhk.edu.cn

Tel.: (86) 755-2351-9515

Hongli Hu

School of Life and Health Sciences, the Chinese University of Hong Kong, Shenzhen, Tu H.L. building, 7^th^ Floor, Longxiang Road, Shenzhen, Guangdong 518172, China.

Email: honglihu@cuhk.edu.cn

Tel.: (86) 755-2351-9007

**Supplementary Text**

Examination of TGR5 residues for 23H recognition

L71A^2.60^ affected agonist potency by one order of magnitude, indicating hydrophobic interaction with 23H. Bulky side-chain substitutions of A217P^6.28^ and C236W^6.47^ on TM6 also caused the reduction of agonist potency by one order of magnitude. It is worth mentioning that A217P^6.28^, associated with primary sclerosing cholangitis, is located on the interface between TGR5 and G_as_. For other substitutions with significant cAMP signal decreasing, such as N93A/F^3.33^, F161A^5.35^, and Y165A^5.39^, the expression level dramatically dropped lower than 20% compared to wild type TGR5 in cell surface (supplementary Fig. 6)

**Material and Methods**

**Construction and expression of TGR5**

For TGR5 expression, wild type Homo sapiens TGR5 (NM_001077191.2), truncating from T6 to S307, was cloned into pFastbac vector. To increase protein expression level and stability, an N-terminal FLAG epitope, a 3C protease site, and BRIL fusion protein were inserted after a HA signal peptide. The construct was expressed in sf9 insect cells using the Bac-to-Bac system. Cells were infected at a density of 4×10^6^ cells per ml and expressed at 27 ℃ for 48 hours. For cAMP assays, the full-length TGR5 with indicated point mutants were cloned into mammalian expression vector pCDNA 3.1 with an N-terminal Flag tag and a 3C protease site inserted after a HA signal peptide.

**Construction, expression and purification of G_s_ heterotrimer and Nb35**

Gs heterotrimer was expressed in High Five insect cells (Invitrogen). Human G_αs_ was cloned in pFastbac vector, and N-terminal 6×His-tagged rat G_β1_, and bovine G_γ2_ were cloned into pFastBac-Dual vector, and the virus was prepared using Bac-to-Bac baculovirus system. The cells were infected with both G_αs_ and G_βγ_ virus at a ratio of 10:1 at 27 °C for 48 hours. G**_s_** heterotrimer was purified as previously reported. Nanobody-35 (Nb35) was expressed in the E. *coli* BL21 (DE3), extracted, and purified by general nickel affinity chromatography protocol as a small soluble protein.

**TGR5-G_s_-Nb35 complex formation and purification**

TGR5 agonist 23H is a kindly gift from Shen Lab in Shanghai Institute of Materia Medica, Chinese Academy of Sciences. 1 L Sf9 cell pellets infected with virus containing TGR5 were lysed in 60 ml lysis buffer 10 mM HEPES, pH 7.5, 1 mM EDTA, 4 mg/ml iodoacetamide, 2.5 μg/ml leupeptin, 0.16 mg/ml Benzamidine, 0.46 μM agonist 23H. The cell membrane was collected by centrifuge at 186,000g. 10 mg of G_s_ and 1.6 mg Nb35 were added and incubated at RT for 1 hr. 10 mM MgCl_2_ and apyrase was added, incubated at RT for additional 30 mins. The protein complex was formed on the membrane and then 1% LMNG (NG310 Anatrace), 0.1% CHS (CH210, Anatrace) was added to solubilize the membrane. Extracted protein complex was further purified in two steps, by Ni-NTA resin affinity chromatography and Flag M1 resin affinity chromatography. During wash steps, the buffer was exchanged to 25 mM HEPES, pH 7.5, 150 mM NaCl, 0.06% LMNG, 0.006% CHS, 0.02% GDN (GDN101, Anatrace), 100 μM TCEP, 10% glycerol, 0.46 μM agonist 23H. The protein complex was eluted by elution buffer 25 mM HEPES, pH 7.5, 150 mM NaCl, 0.006% LMNG, 0.0006% CHS, 0.002% GDN, 100 μM TCEP, 0.46 uM agonist 23H, 5 mM EDTA, 200 uM flag peptides. Protein complex was concentrated and loaded onto Superose 6 increase 10/300 GL column with running buffer 25 mM HEPES, pH 7.5, 150 mM NaCl, 0.006% LMNG, 0.0006% CHS, 0.002% GDN, 100 μM TCEP, 0.46 μM agonist 23H. Fractions contains TGR5-G_s_ complex was collected and concentrated for electron microscopy experiments.

**Cryo-EM sample preparation and data collection**

The purified protein complex was concentrated to about 8 mg/ml for cryo-sample preparation. The amorphous alloy film (CryoMatrix Au300- R1.2/1.3, Zhenjiang Lehua Electronic Technology Co., Ltd.) was glow discharged with air for 40 sec at 10 mA at PELCO easiGlow™ Glow Discharge Cleaning System. Aliquots of 3.5 μl the protein complex was placed on the gird, and then was blotted for 3.5 sec and flash-frozen in liquid ethane cooled by liquid nitrogen with Vitrobot (Mark IV, Thermo Fisher Scientific). For data collection, the 300 kV Titan Krios Gi3 equipped with Gatan K3 Summit detector and GIF Quantum energy filter was operated. Movie stacks with 40 frames were automatically collected using EPU (Thermo Scientific) at a nominal magnification of 105,000X, corresponding to a pixel size of 0.85 Å. The defocus range was set from -1.5 μm to -2.5 μm. Each stack was exposed in the counted-Nanoprobe mode for 2.48 sec and the total dose rate was about 50 e^-^/Å^2^ for each stack.

**Cryo-EM image processing**

Dose-fractionated movie stacks were subjected to dose-weighted motion correction by MotionCor2 v1.2.2 using 5×5 patches^1^. Contrast transfer function parameters were estimated by Gctf v1.06^2^. Micrographs with weak information were thrown away and 8194 images were chosen for further process. The particles auto-picking, 2D and 3D classification, 3D refinement were performed using RELION 3.0^3^. The total number of 3,683,494 particles were extracted and subjected to a reference-free 2D classification. The selected particles from 2D classification were further subjected to 3D classifications with initial model of a known GPCR-G_s_ complex with EMDB ID 8653 and lowpass-filtered to 30 Å. Eventually, a subset of 450,271 particles were selected from 3D classification were subjected to 3D refinement by subtracting the density of micelle and alpha helical domain, resulting a final map with global resolution of 3.9 Å at FSC 0.143. Local resolution map was calculated from the Bsoft packageat cutoff FSC of 0.5^4^.

**Model building and refinement**

The initial template of TGR5 was derived using Modeller^5,6^ from a class A GPCR (PDB 6CMO). Sequence alignment was done using Clustal W2.1^7^. The coordinates of Gs and Nb35 from PDB 5VAI^8^ were used as initial models. Models were docked into the EM density map using UCSF Chimera^9^, followed by iterative manual building in Coot^10^. The final model was subjected to real space refinement in Phenix^11^. *Molprobity* was used to evaluate model geometry.

**Computational docking**

Computational docking of ligand 23H to TGR5 receptor cryo-EM structures was performed using AutoDock Vina. A 25×25×25 Å^3^ box centered at the observed density map corresponding to the ligand was selected as the searching region, which is large enough to cover the whole binding pocket of TGR5. Flexibility of the ligand and all side-chain of the residues within the searching region were considered. Exhaustiveness (80) docking was carried out to search the optimized ligand binding pose, with the so-called Broyden-Fletcher-Goldfarb-Shanno (BFGS) algorithm for local optimization. Three representative docking poses were selected and fitted into the EM map using Chimera.

**cAMP assay**

cAMP accumulation after TGR5 activation was analyzed by a time-resolved fluorescence resonance energy transfer (TR-FRET) technology. TGR5 constructs were transfected into HEK293 cells with Lipofectamine 3000 reagent (Thermo Fisher Scientific; Waltham, MA, USA). After 24 h transfection, the cells were harvested in an assay buffer (HBSS plus 0.5 mM isobutylmethylxanthine, 0.1% BSA (w/v), and 5 mM HEPES) and transferred to a 384-well plate. Various ligands were prepared as a series of eight 10-fold serial dilutions and applied to stimulate the cells in a cell incubator for 30 min. The cAMP levels in the stimulated cells were detected with a cAMP detection kit (PerkinElmer; ‎Waltham, MA, USA) according to the manufacturer’s instructions. The measurement was conducted with Victor Nivo plate reader (PerkinElmer). cAMP concentrations were calculated according to TR-FRET signals of cell samples and cAMP standards. Basal cAMP levels were subtracted and the cAMP responses were shown as percentages of the maximal response. The data were analyzed with a GraphPad Prism 5.0 software (GraphPad Inc, La Jolla, CA, USA).

**TGR5 constructs expression level determination by flow cytometry**

Approximately 1×10^6^ transfected HEK293 cells were collected and washed with PBS. Cells were blocked with 5% BSA at room temperature for 15 min and then incubated with anti-FLAG antibody (1:100) in PBS containing 1% BSA at 4°C for 1 h. Cells were washed three times with PBS and then incubated with anti-mouse Alexa-488-conjugated secondary antibody (1:300, Beyotime) in PBS containing 1% BSA at 4 °C in the dark. Washed two times with PBS and then the cells were resuspended in 200 μl PBS for detection in BD Accuri™ C6 Plus flow cytometer. Approximately 10,000 cellular events were counted for each sample and the fluorescence intensity data were collected.

**Supplementary References**

Zheng,

**Figure Legends：**


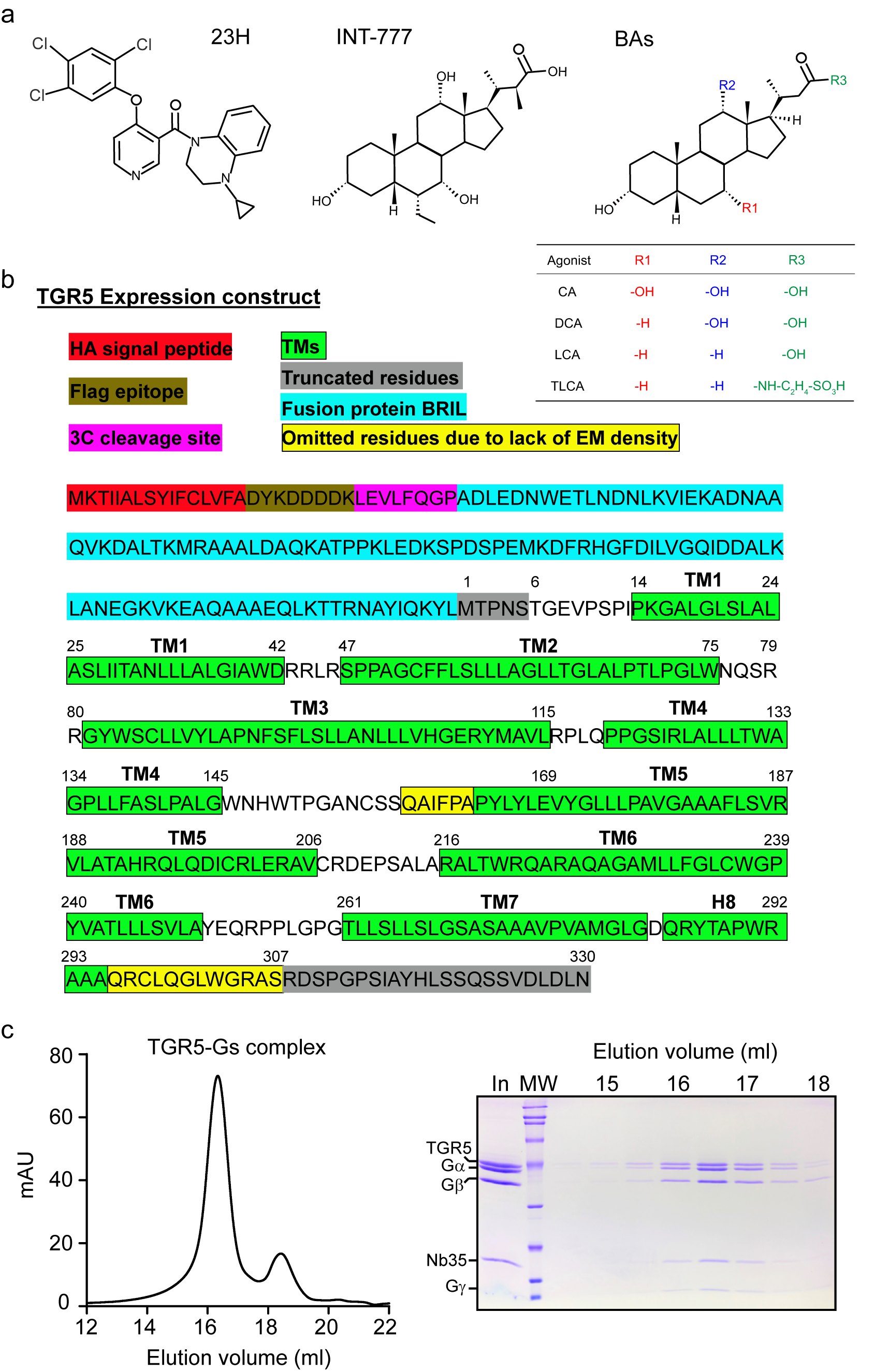


**Fig. S1 | Amino acid sequence of the TGR5 construct and the purification of the TGR5-G_s_ complex used for determination of structure. a,** Structural formula of non-steroid compound 23H, semi-synthetic compound INT777 and four bile acids (BAs). **b**, The sequences are annotated to denote the location of the haemagglutinin (HA) signal sequence (red highlight), Flag (brown highlight), 3C cleavage sites (magenta highlight), fusion protein (cyan highlight), truncated residues (grey highlight). Transmembrane helical domains in TGR5 are boxed and highlighted in green. Segments of the TGR5 that were not resolved in the cryo-EM map are highlighted in yellow. **c**, Left panel shows the final size exclusion chromatography elution profile of the TGR5-G_s_ complex with 23H. Right panel shows the SDS–PAGE and Coomassie blue staining of the size-exclusion chromatography peak, demonstrating the presence of each of the components of the complex.


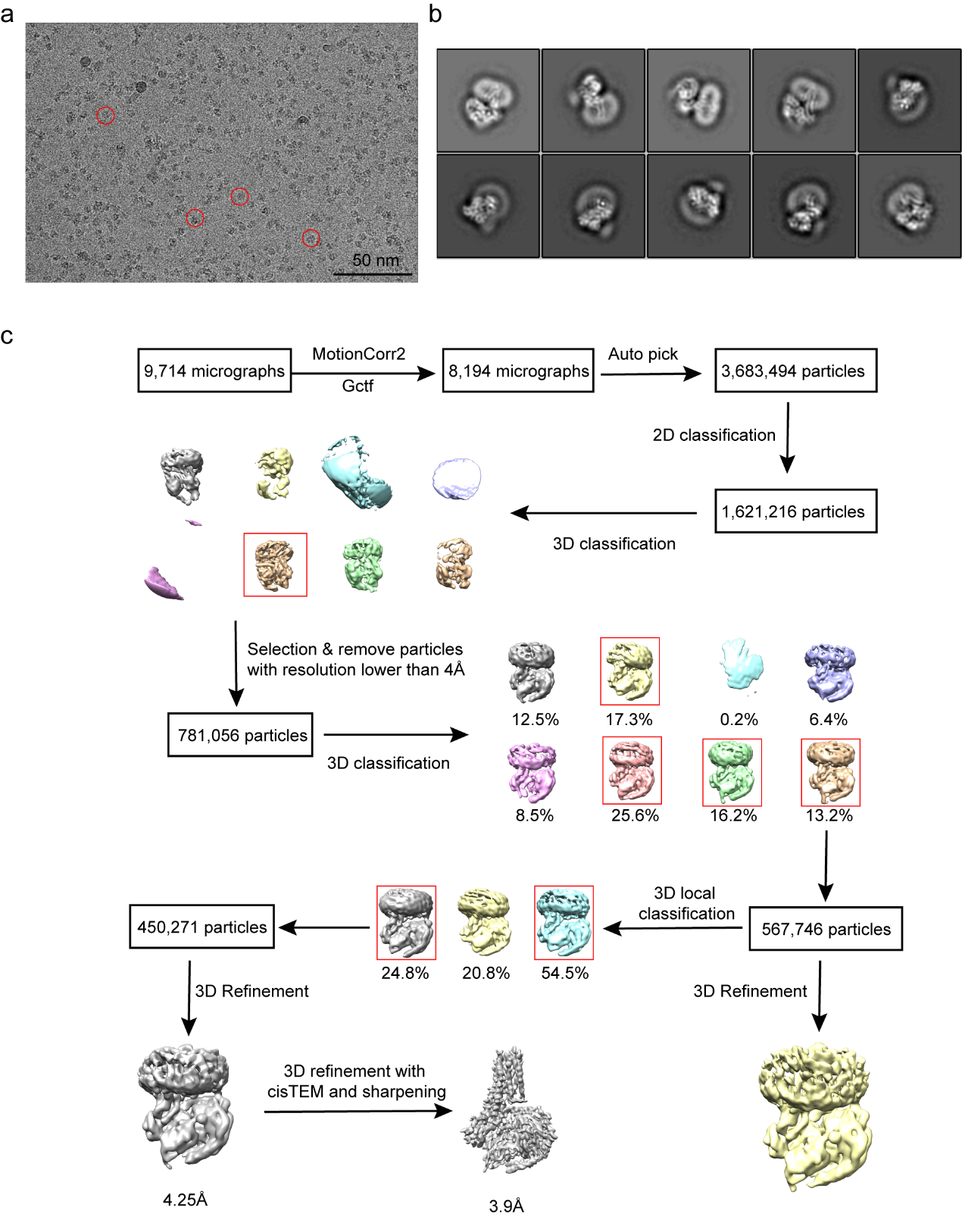


**Fig. S2 | Cryo-EM micrograph, 2D class averages, and single-particle cryo-EM analysis of the TGR5-G_s_ complex.** **a**, Cryo-EM micrograph of the activated TGR5-G_s_ complex. Examples of particle projections are circled. Scale bar, 50 nm. **b**, Representative 2D averages show distinct secondary structure features for TGR5-G_s_ complex. **c**, Flow chart of cryo-EM data processing of the TGR5-G_s_ complex. Details are provided in the Methods section.


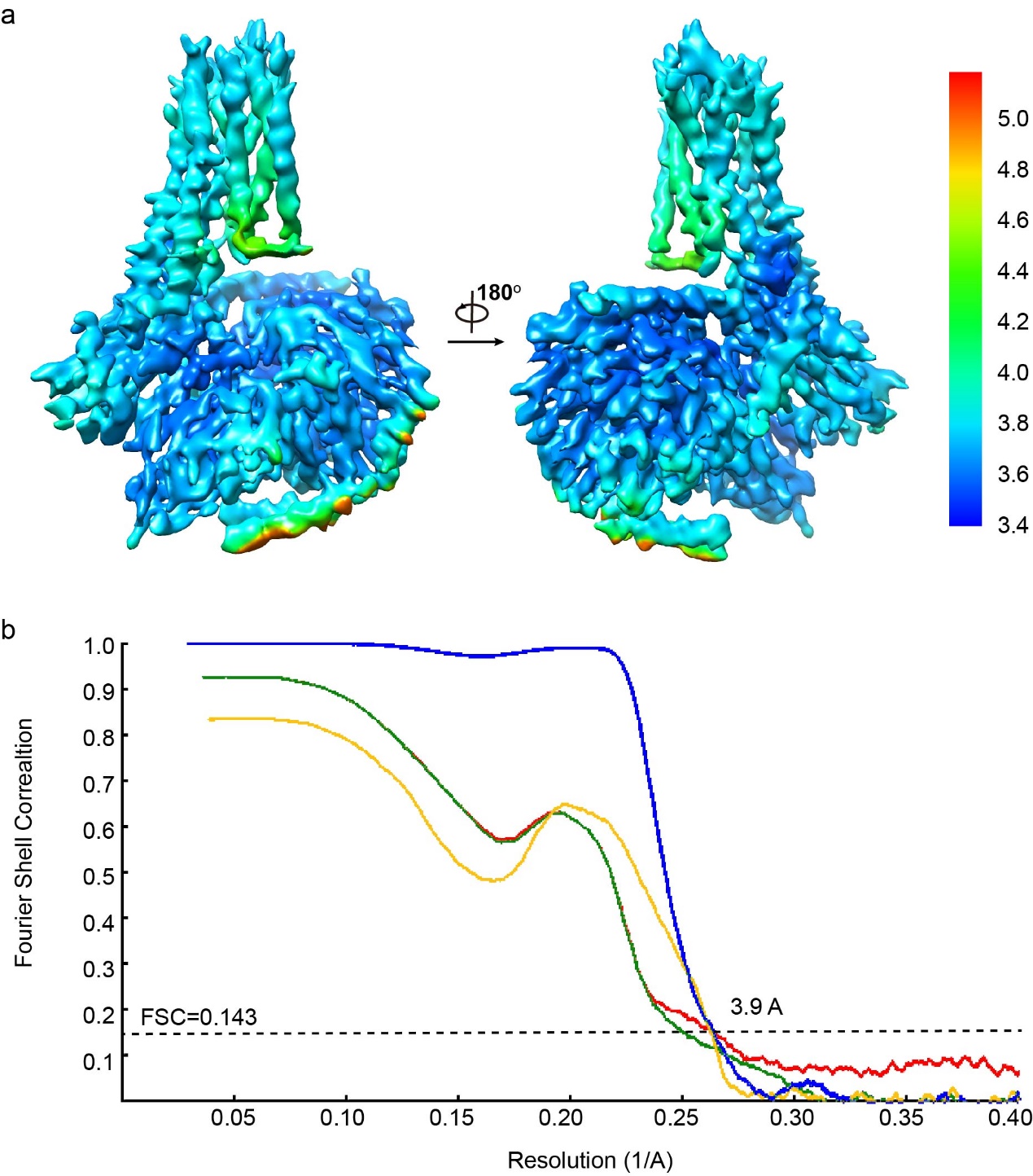


**Fig. S3 | Resolution of cryo-EM map and validation of the TGR5-G_s_ structure.** **a**, Final three-dimensional density map colored according to local resolution. **b,** Resolution estimation of the EM map. Gold standard Fourier shell correlation (FSC) curve (blue) and cross-validation of model to cryo-EM density map. Model overfitting was evaluated through its refinement against one cryo-EM half map after randomly displacing all atoms by 0.2 Å. FSC curves were calculated between the resulting model and the half map used for refinement (red), as well as between the resulting model and the other half map for cross validation (green), and also against the full map (yellow).

**
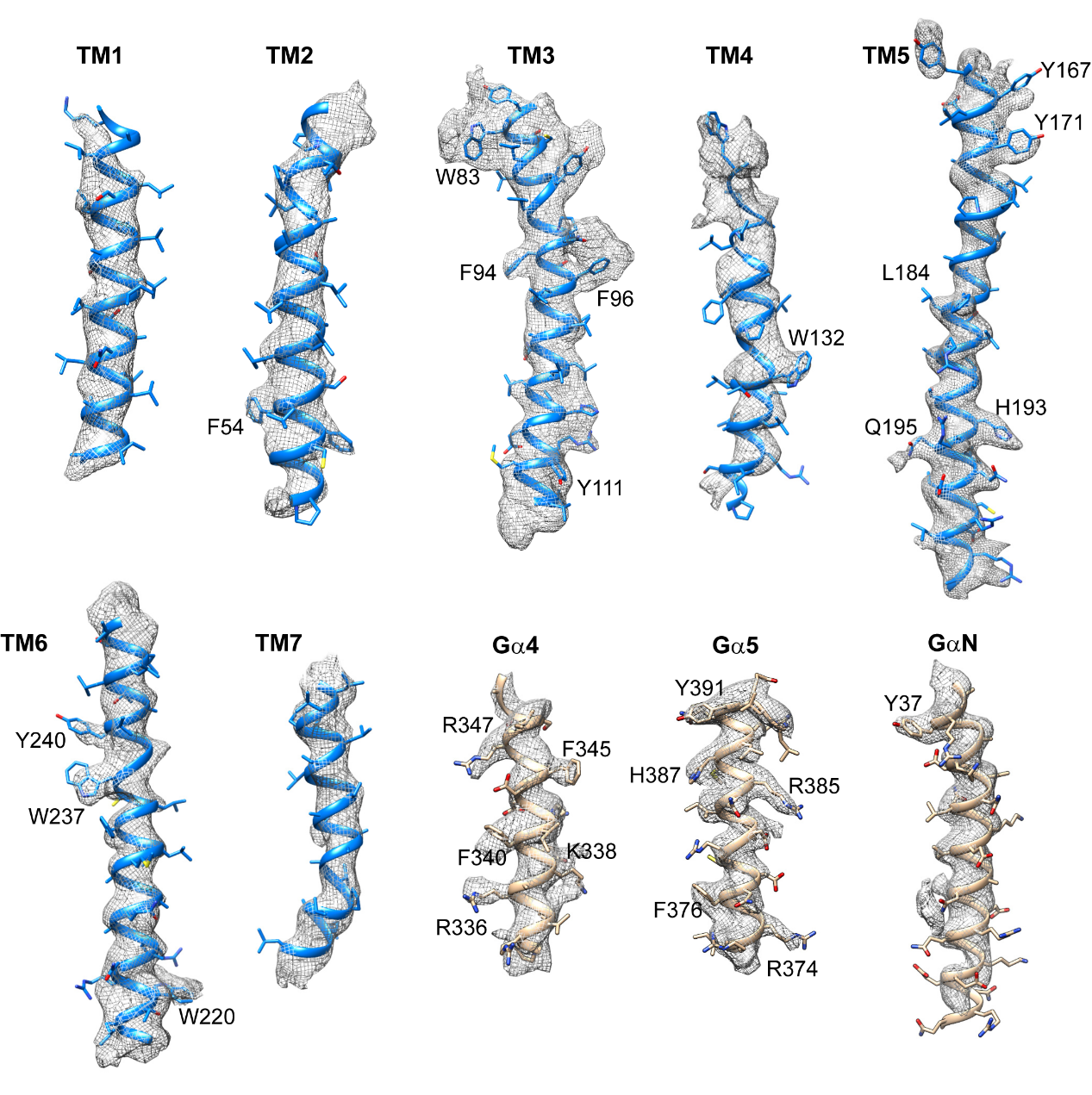
**

**Fig. S4 | Representative Cryo-EM map for key components of TGR5-G_s_ complex.** Cryo-EM density map and model are shown for all seven transmembrane α-helices and the α4-, α5-, and αN-helices of the G_s_ protein. Bulky resides are indicated for each segment.


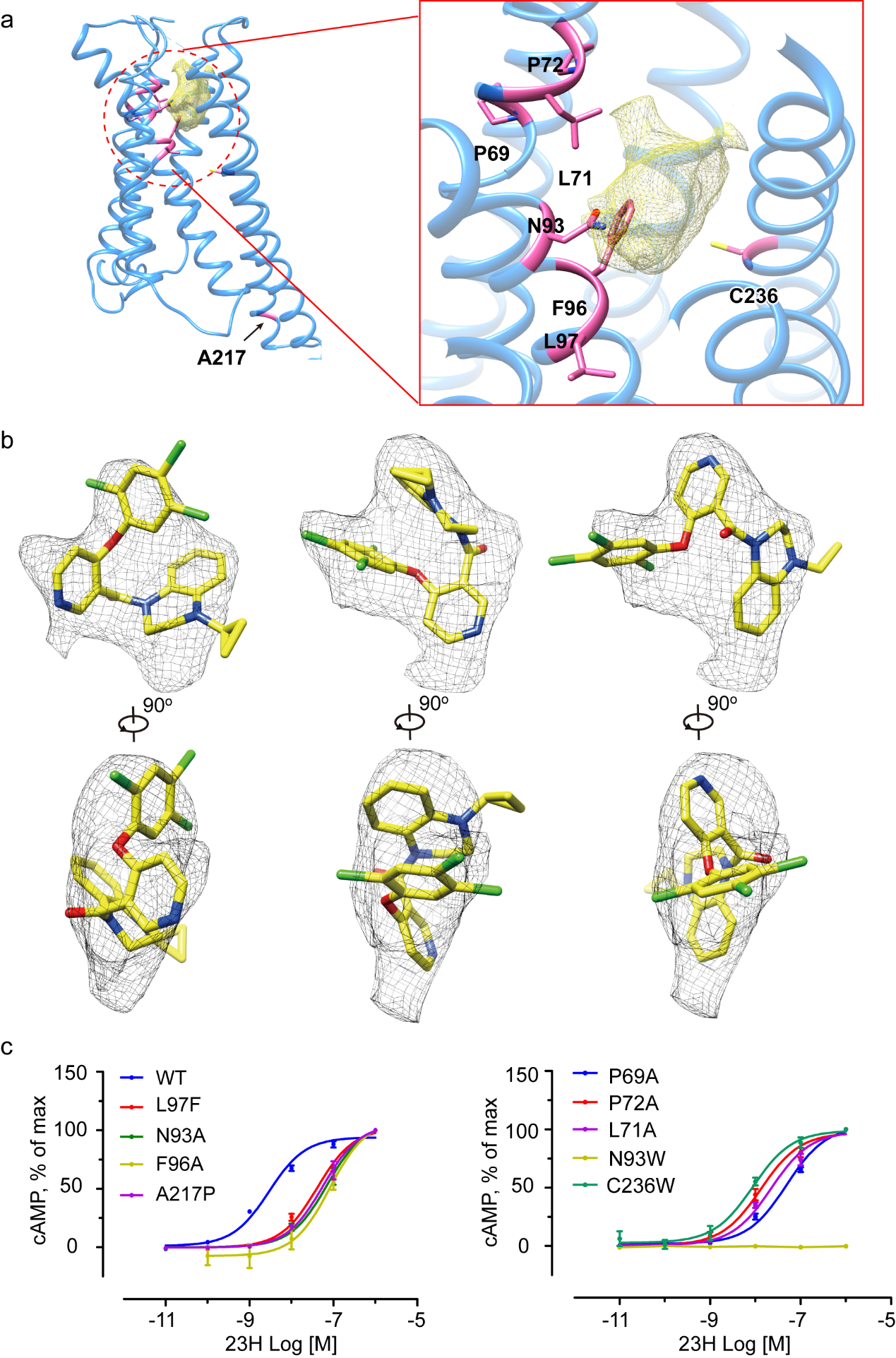


**Fig. S5 | cAMP response of mutant TGR5 with 23H.** **a,** Residues in TGR5 that involve in 23H binding. Density of 23H is shown in yellow. Residues involve in 23H binding are shown in pink. **b**, Three possible binding models for 23H. Two perpendicular views are shown. The black mesh represented the omit map in the putative agonist binding pocket. **c,** cAMP responses of mutant TGR5. These mutations of TGR5 reduced agonist potency by one order compared with wild type. cAMP responses are shown as percentages of the maximum response of each mutation or the WT (for N93W with significant reduction in efficacy). The corresponding pEC50 is shown in Extended Data Table 2. WT data was not shown on panel b (right) because all the mutations were tested at the same time. The data represent means±*S.E.M.* (n=3-5).


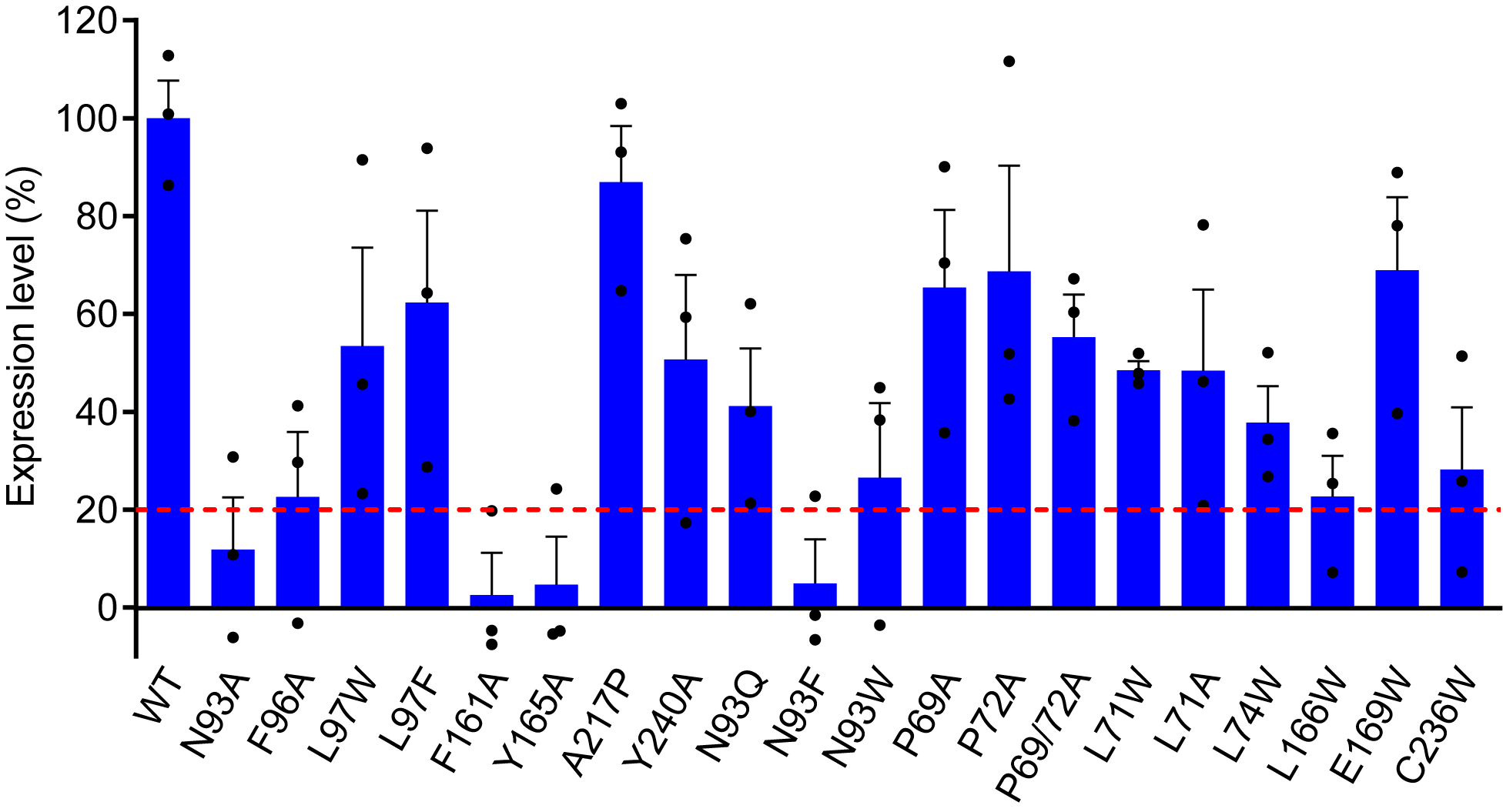


**Fig. S6 | Expression level of mutational TGR5 in cells by quantitative flow cytometry.** Red dashed line indicates 20% expression level of wild type TGR5. The data are shown as means±*S.E.M.* of three independent experiments.

**
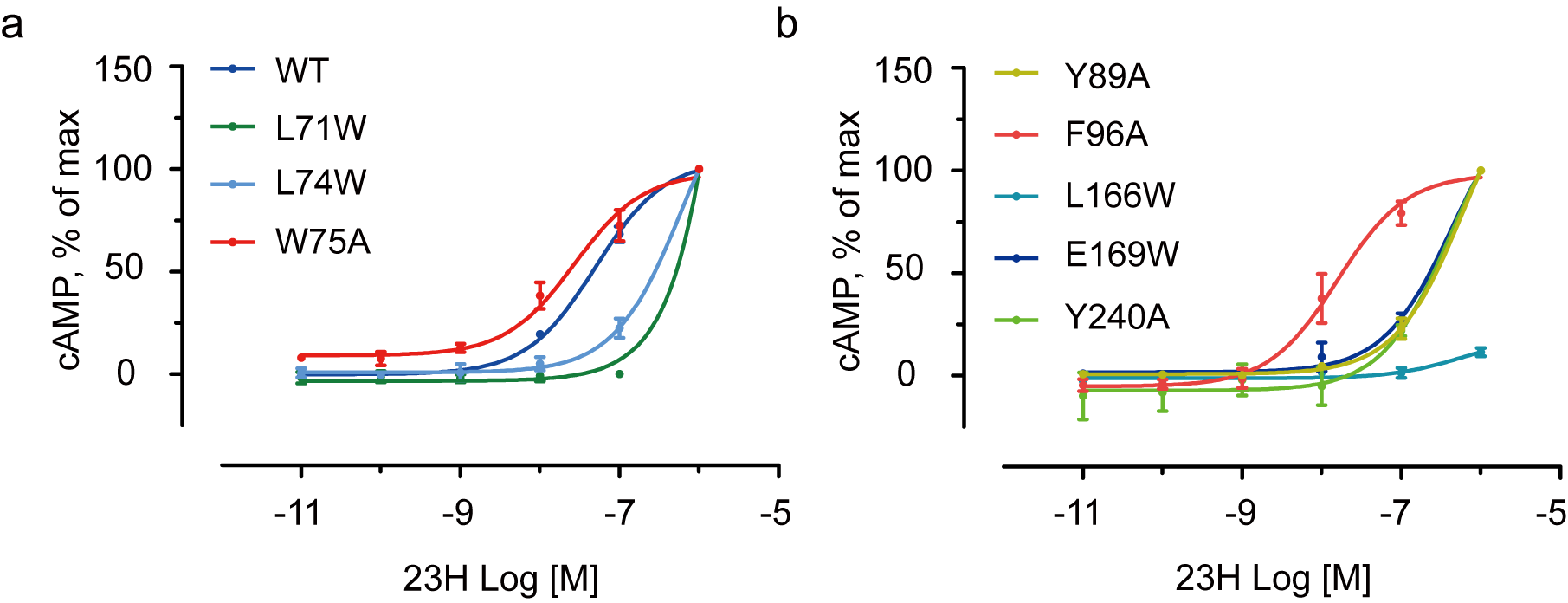
**

**Fig. S7 | cAMP responses of mutant TGR5 to LCA.** cAMP responses are shown as percentages of the maximum response of each mutation or the WT (for mutations with significant reduction in efficacy). The corresponding pEC50 is shown in Extended Data Table 3. WT data was not shown on panel b because all the mutations were tested under the same experimental conditions. The data represent means±*S.E.M.* (n=3-5).


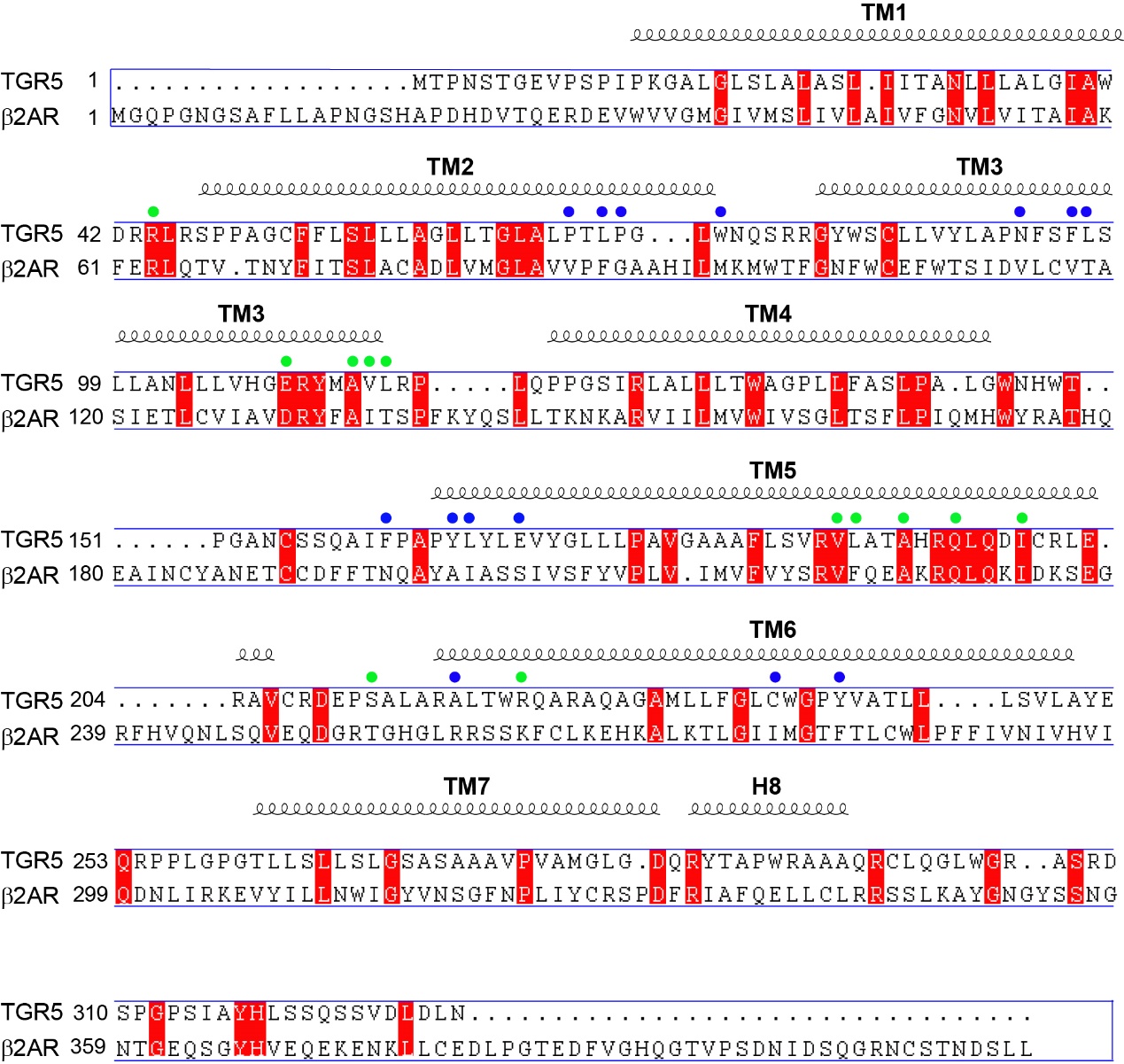


**Fig. S8 | Sequence alignment of TGR5 and β_2_AR.** The alignment was performed using MultAlin and ENDscript programs. Secondary structural elements are shown above the sequence. Sequence identity is shown in white letters on a red background. The blue dots indicate residues that were mutated to verify compound 23H interaction (in Fig. 1c-d and Extended Data Fig. 5). The green dots indicate the residues in TGR5 that interact with G_s_ protein (in Fig. 1f). Some of these residues are identical to that of β_2_AR which involve in G_s_ protein binging.


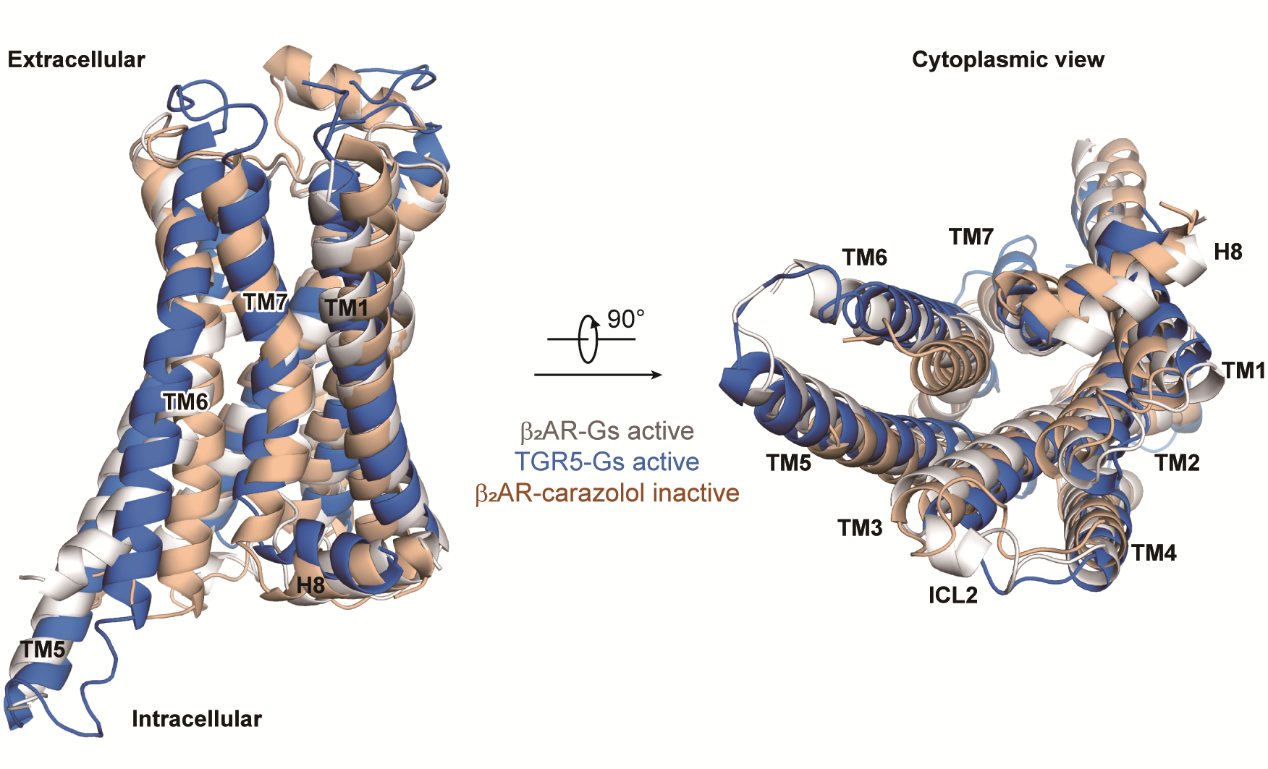


**Fig. S9 | Comparisons of active TGR5 with active and inactive β_2_AR.** Active TGR5, inactive and active β_2_AR were shown, by two perpendicular views, in marine, wheat and gray cartoon models, respectively.


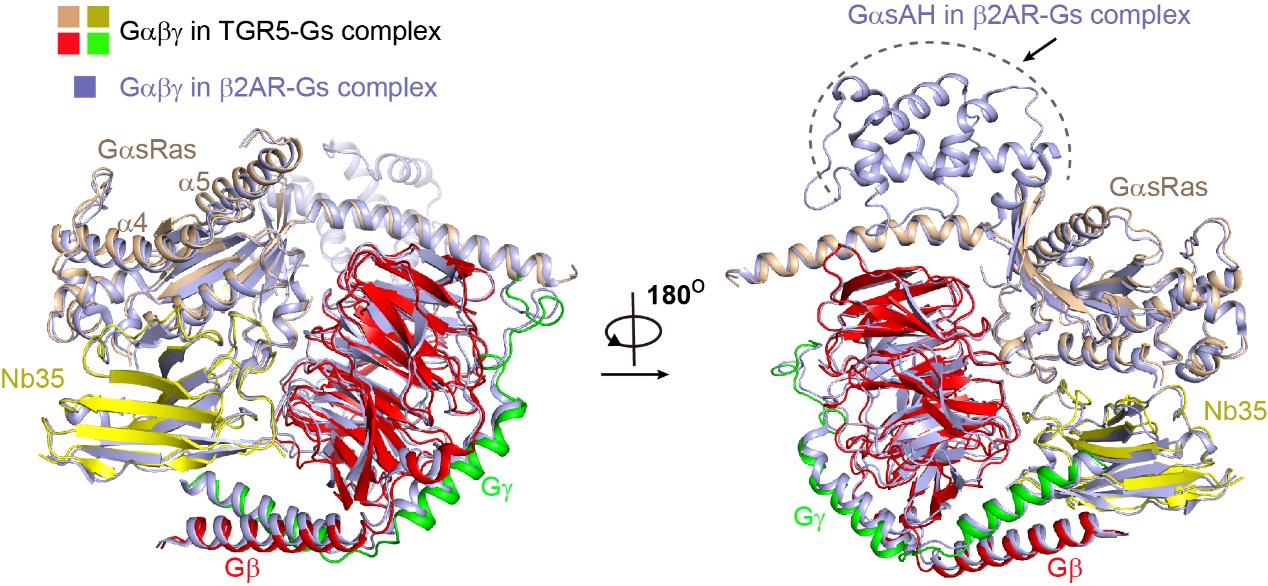


**Fig. S10 | Comparison of G_s_ proteins in structures of TGR5-G_s_ complex and** **β_2_AR-G_s_ complex.** G_αβγ_ complex and Nb35 in β_2_AR-Gs complex are shown in light blue. G_αs_Ras, G_β_, G_γ_, and Nb35 in TGR5-G_s_ complex are shown in wheat, red, green, and yellow. G_αs_AH domain in β_2_AR-Gs complex is invisible due to the limited electron density.

**Table S1** **| Data collection, phasing and refinement statistics (molecular replacement)**

| **Protein** | **TGR5-G_s_-Nb35**  **EMDB code: EMD-30221**  **PDB code: 7BW0** |
| --- | --- |
| **Cryo-EM Data Collection** |  |
| Voltage (kV) | 300 |
| Magnification (x) | 58,823 |
| Pixel size (Å) | 0.85 |
| Electron exposure (e^-^/ Å^2^) | 50 |
| Defocus range (μm) | [-2.5, -1.5] |
| Number of image stacks | 9714 |
| Number of frames per stack | 40 |
| **Cryo-EM Data Processing** |  |
| Initial number of particles | 3,683,494 |
| Final number of particles | 450,271 |
| Map sharpening B factor (Å^2^) | -230 |
| Map resolution (Å) | 3.9 |
| Map resolution range (Å) | 3.4-7.7 |
| FSC threshold | 0.143 |
| **Model Refinement** |  |
| Model resolution range  Number of amino acids | 3.5-9  1031 |
| Total non-hydrogen atoms | 7946 |
| Bond length r.m.s.d. (Å) | 0.004 |
| Bond angle r.m.s.d. (°) | 1.016 |
| Ranmachandran Plot |  |
| Favored (%) | 93.79 |
| Allowed (%) | 6.21 |
| Outliers (%) | 0.00 |
| Rotamer outliers (%) | 0.12 |
| MolProbity score | 1.98 |

**Table S2 | The potencies of TGR5 ligands with the receptor or its mutants.** The data represent means±*S.E.M.* (n = 3-5).

| pEC50 in  Fig. 1a | | pEC50 in  Fig. 1d | | pEC50 in  Extended Data Fig. 5 | | pEC50 in  Extended Data Fig. 7 | |
| --- | --- | --- | --- | --- | --- | --- | --- |
| Ligands  (TGR5 WT) | pEC_50_±SEM | TGR5 WT/mutations | pEC_50_±SEM | TGR5 mutations | pEC_50_±SEM | TGR5 mutations | pEC_50_±SEM |
| 23H | 8.4±0.13 | WT | 8.39±0.13 | P69A | 7.23±0.09 | L71W | No response |
| INT777 | 5.83±0.12 | P69/72A | 6.33±0.33 | L71A | 7.63±0.12 | L74W | 4.17±0.25 |
| CA | 4.97±0.12 | L71W | 6.20±0.29 | P72A | 7.83±0.12 | W75A | 5.50±0.29 |
| LCA | 5.63±0.17 | N93Q | 6.53±0.17 | N93A | 7.13±0.24 | Y89A | 4.13±0.29 |
| TLCA | 6.13±0.12 | L97W | 6.23±0.17 | N93W | 6.97±0.12 | F96A | 6.03±0.26 |
|  |  | L166W | 5.80±0.24 | F96A | No response | L166W | No response |
|  |  | E169W | 6.27±0.12 | L97F | 7.37±0.12 | E169W | 4.33±0.12 |
|  |  | Y240A | 6.00±0.14 | A217P | 7.23±0.24 | Y240A | 4.37±0.12 |
|  |  |  |  | C236W | 8.00±0.14 |  |  |
